# Genome-wide identification of *PME* genes, evolution and expression analyses in soybean (*Glycine max* L.)

**DOI:** 10.1186/s12870-021-03355-1

**Published:** 2021-12-06

**Authors:** Liang Wang, Yingqi Gao, Songming Wang, Qiqi Zhang, Shouping Yang

**Affiliations:** Soybean Research Institute, National Center for Soybean, Key Improvement Laboratory of Biology and Genetic Improvement of Soybean (General, Ministry of Agriculture), State Key Laboratory of Crop Genetics and Germplasm Enhancement, Jiangsu Collaborative Innovation Center for Modern Crop Production, College of Agriculture, Nanjing Agricultural University, Nanjing, 210095 China

**Keywords:** Soybean, *PME*, Genome-wide identification, Evolution, Expression analyses, Flower bud development, Seed germination

## Abstract

**Background:**

Pectin methylesterase (PME) is one of pectin-modifying enzyme that affects the pectin homeostasis in cell wall and regulates plant growth and diverse biological processes. The *PME* genes have been well explored and characterized in different plants. Nevertheless, systematic research on the soybean (*Glycine max* L.) *PME* genes remain lacking.

**Results:**

We identified 127 *Glycine max PME* genes (*GmPME*) from the soybean *Wm82.a2.v1* genome, which unevenly distributed on 20 soybean chromosomes. Phylogenetic analysis classified the *GmPME* genes into four clades (Group I, Group II, Group III and Group IV). *GmPME* gene members in the same clades displayed similar gene structures and motif patterns. The gene family expansion analysis demonstrated that segmental duplication was the major driving force to acquire novel *GmPME* genes compared to the tandem duplication events. Further synteny and evolution analyses showed that the *GmPME* gene family experienced strong purifying selective pressures during evolution. The *cis*-element analyses together with the expression patterns of the *GmPME* genes in various tissues suggested that the *GmPME* genes broadly participate in distinct biological processes and regulate soybean developments. Importantly, based on the transcriptome data and quantitative RT-PCR validations, we examined the potential roles of the *GmPME* genes in regulating soybean flower bud development and seed germination.

**Conclusion:**

In conclusion, we provided a comprehensive characterization of the *PME* genes in soybean, and our work laid a foundation for the functional study of *GmPME* genes in the future.

**Supplementary Information:**

The online version contains supplementary material available at 10.1186/s12870-021-03355-1.

## Background

The dynamics of cell walls such as constructions, differentiations, maturations and degradations are crucial for regulating plant growth [1, 2]. Pectin is one of the most abundant components of plant cell walls, which is associated with the porosity and hydration of the cell wall as well as the plant cell intercellular adhesion [3, 4]. Homeostasis of pectin is balanced by different pectin-regulating enzymes [5, 6]. Pectin methylesterase (PME, EC 3.1.1.11), so-called pectinesterase, is a hydrolytic enzyme that catalyzes the de-esterification of methyl esterified galacturonic acid residues of pectin to generate carboxyl groups and release methanol and hydrogen ions in the cell wall [7, 8]. A high level of PME often induces the loosening of the cell wall, whereas, low PME activity tends to form a rigid cell wall [2]. Early studies turned out that the PME activity was tightly linked to distinct plant developmental courses, including pollen tube elongation [9, 10], seed germination [11, 12], hypocotyl growth [13, 14], fruit ripening and softening [2, 15, 16].

In recent years, whole-genome sequencing technologies promoted the exploration of multiple gene families in diverse species [17]. Genome-wide identifications and characterizations of *PME* genes have been carried out in diverse plant species, including Arabidopsis [18], rice [19], maize [20], cotton [21], tomato [16] and strawberry [2]. For the gene structures, all the *PME* gene members contain the PME domains, and some of them meanwhile harbor the PME inhibitor (PMEI) domains [21]. For the gene functions, different *PME* genes play multiple roles in plants. In Arabidopsis, *AtPME3* was reported to affect the metabolism of seed storage proteins during the post-germinative growth of seedlings [22]. *AtPME17* was another functional pectin methylesterase gene, which was regulated by its PRO region that triggered PME activity in the resistance to *Botrytis cinerea* [23]. In another study, *AtPME34* was turned out to contribute to heat tolerance by promoting stomatal movement in Arabidopsis [5]. Besides, *Brassica campestris Male Fertility 23a* (*BcMF23a*) was an essential pectin methylesterase gene that proved to be required for microspore development and pollen tube growth in *Brassica campestris* [24]. And *BcPME37c* is another published pectin methylesterase gene that was involved in the pollen intine formation in *Brassica campestris* [25].

Soybean (*Glycine max* L.) is an important economic crop rich in high-quality plant protein and oil [26]. The latest research carried out identification and expression analyses of the *PMEI* genes throughout the soybean genome [27]. However, the systematic investigation of the *Glycine max PME* gene (*GmPME*) family in soybean remains blank. In this study, we comprehensively identified 127 *GmPME* genes in the soybean genome. According to the classification of the *PME* gene members in Arabidopsis, we phylogenetically clustered and divided the GmPME members into four clades (Group I, Group II, Group III, and Group IV). Then we explored the gene structures, motif patterns, chromosomal locations and gene duplication events of the identified GmPME members. Moreover, we conducted the evolutionary analyses on the PME members among soybean and representative dicotyledons as well as monocotyledons. To survey the potential roles of *GmPME* genes in regulating various biological processes, we analyzed the *cis*-elements in the promoter regions of the *GmPME* genes and gene expression profiles in different tissues. High-yielding is one of the most important targets in soybean breeding [28], utilization of heterosis with the cytoplasmic male sterility (CMS) system is an effective way to fulfill this goal [29]. At present, stable soybean CMS lines, such as NJCMS1A, NJCMS2A and NJCMS5A, have been well developed and applied for soybean hybrid seed production [30]. Hence, we conducted *GmPME* gene expression analyses between stable soybean cytoplasmic male sterile line NJCMS1A and its maintainer NJCMS1B. Importantly, a good seed germination ability is also essential for soybean production [31]. In the current research, we investigated the expression patterns of the identified *GmPME* genes during seed germination processes. Besides, 17 representative *GmPME* genes were selected and manipulated further quantitative RT-PCR analyses during soybean flower bud development and seed germination. Overall, the current study provided a systematic characterization of the GmPME members. To a certain degree, our work facilitated future relevant gene functional research and may play a fulfilling role in promoting soybean high-yielding breeding.

## Results

### Identification of soybean *PME* genes

From the soybean *Wm82.a2.v1* genome on Phytozome (https://phytozome-next.jgi.doe.gov/), we identified 127 *GmPME* genes originated from 20 different soybean chromosomes. Based on the chromosome orders and gene chromosomal locations, the 127 *GmPME* genes were designated as *GmPME1* to *GmPME127*, respectively. (Additional file 1: Table S1).

The fundamental information of GmPME members was displayed in Table S1 (Additional file 1), including the opening reading frame (ORF) length, the protein size (aa, amino acid), the isoelectric point (pI), the molecular weight (MW), the predicted subcellular localization and the conserved domain. As is illustrated in Table S1 (Additional file 1), the protein sizes of GmPME members were ranged from 107 aa (GmPME92) to 1186 aa (GmPME54). Accordingly, the MW of GmPME members spanned from 11,780.63 Da to 129,972.9 Da, and ORF length varied from 321 bp to 3558 bp. Besides, the pI of the GmPME members ranged from 4.61 (GmPME92) to 10.12 (GmPME82). For the subcellular locations, 68 GmPME members were predicted to be located in the extracellular region, 38 in the plasma membrane, 15 were in the cytoplasmic region, three were in the nuclear region, two were in the mitochondrial region and one was in the chloroplast. And the gene coding sequences and protein sequences of the GmPME members were listed in Table S2 (Additional file 2).

### Phylogenetic classifications of GmPME members

**Fig. 1:**
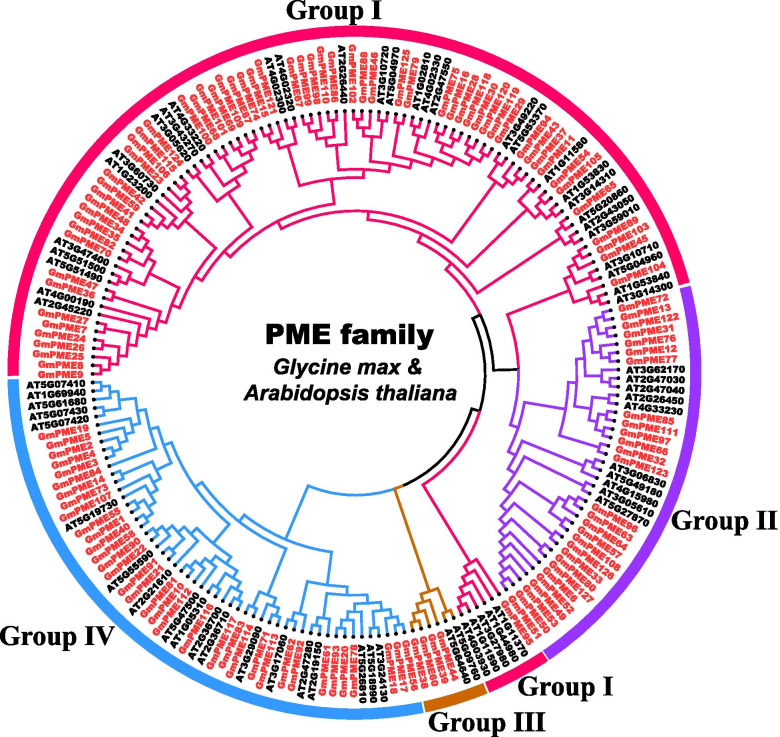
Unrooted phylogenetic tree displaying the relationships among PME proteins between soybean and Arabidopsis. The tree was divided into four hereditary clades (Group I, Group II, Group III and Group IV) with different colored arcs. The prefixes ‘At’ and ‘Gm’ refer to Arabidopsis and soybean, respectively. All GmPME proteins were highlighted in red

Referring to the classification of *Arabidopsis thaliana* PME (AtPME) members [18], we utilized the 66 published AtPME proteins (Additional file 3: Table S3) and the 127 identified GmPME proteins to construct a phylogenetic tree. Correspondingly, the *GmPME* gene members were divided into four clades: Group I, Group II, Group III and Group IV (Fig. 1). As is shown in Fig. 1 and Table S1 (Additional file 1), there were 60 GmPME members in the Group I clade, 28 in the Group II clade, four in the Group III clade, and 35 in the Group IV, respectively.

### Gene structures and motif patterns of *GmPME* gene members

**Fig. 2:**
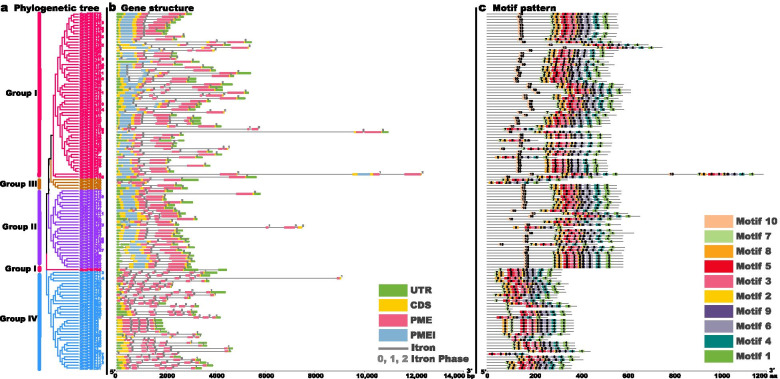
Phylogenetic exhibition of gene structures and motif patterns of the identified GmPME members. **a** Phylogenetic clustering of the *GmPME* gene members. **b** Gene structures of GmPME members. Green boxes are untranslated 5′- and 3′- regions; yellow boxes indicate exons; pink boxes represent PME domains; blue boxes suggest PMEI (PME Inhibitor) domains. The gray lines are introns and the numbers (0, 1, 2) indicate intron phases. **c** Motif patterns of GmPME members. Totally ten distinct MEME-motifs were depicted in different colors, and their sequences were listed in Table S4 (Additional file 4). Besides, the length of relevant gene structures and motif components can be estimated with respective scales at the bottom of panels

The gene structures and motif patterns of *GmPME* gene members were phylogenetically clustered and depicted in Fig. 2. All the identified *GmPME* genes contained the PME domains and different *GmPME* gene members in the same clade tended to display similar gene structures. Interestingly, most *GmPME* gene members in the Group I, Group II and Group III clades simultaneously contained the PMEI domains (except for GmPME27, GmPME35, GmPME95 and GmPME106 in the Group I clade, GmPME96 in the Group II clade, GmPME39 and GmPME44 in the Group III clade) compared to those in the Group IV clade (Fig. 2 and Additional file 1: Table S1). For the exon-intron patterns, there were one to seven exons contained in *GmPME* gene members (one with one exon, 54 with two exons, 23 with three exons, 18 with four exons, 23 with five exons, seven with six exons and one with seven exons), and introns widely existed (Fig. 2b).

**Fig. 3:**
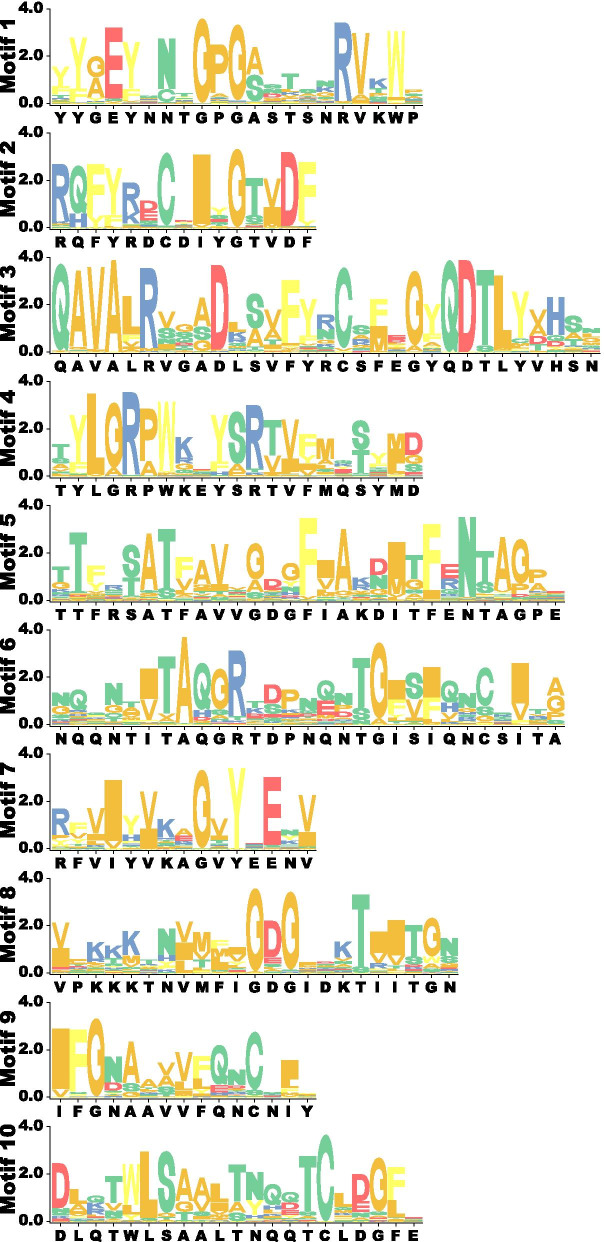
Ten MEME-motif Seq Logos for the GmPME proteins

Meanwhile, the MEME-motif scanning was also carried out to better illustrate the conserved domains of the GmPME members (Fig. 2c). As is shown in Fig. 2c, ten different MEME-motifs (Motif 1 to Motif 10) were acquired. The information of the ten MEME-motifs was listed in Table S4 (Additional file 4) and Seq-Logos of the MEME-motifs were depicted in Fig. 3. Notably, Motif 10 only showed up in those GmPME members that owned the PMEI domains. Hence, we summarized that Motif 10 was corresponding to the PMEI domain, and Motif 1 to Motif 9 were linked with the PME domain in the current study (Additional file 4: Table S4). In general, the MEME-motifs in the specific clades of the *GmPME* gene family showed regular orders and patterns.

### Distributions of *GmPME* genes on soybean chromosomes

**Fig. 4:**
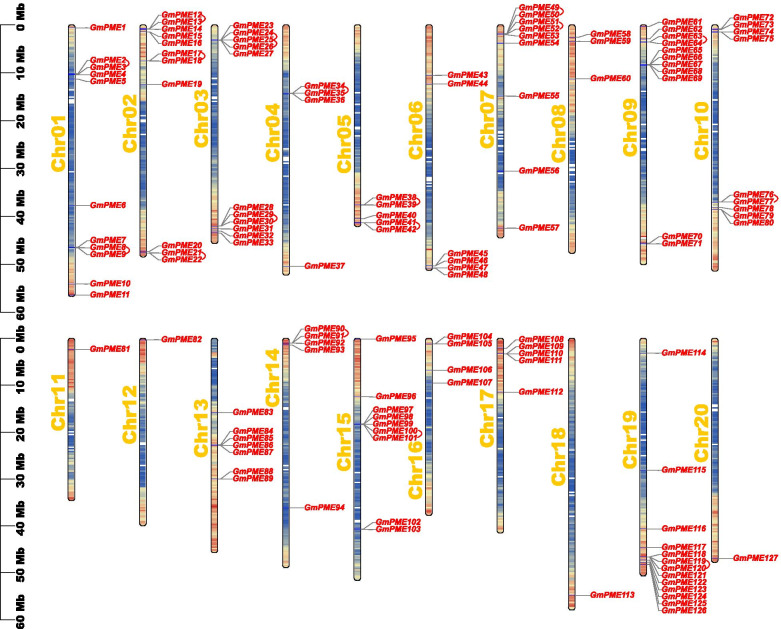
The chromosomal distributions of *GmPME* genes. Soybean chromosomal names were placed at the left of the chromosomes. Gradient colors from red to blue that attached to soybean chromosomes were corresponding from high to low density by setting the estimating hereditary interval as 300 kb. The blank regions on chromosomes were the genetic regions that lacked gene distributing information. Besides, the tandemly duplicated *GmPME* gene pairs were linked by red arcs

The identified *GmPME* genes were physically anchored to the 20 soybean chromosomes (Chr 01 to Chr 20) in Fig. 4. Overall, the *GmPME* genes are unevenly distributed on the chromosomes throughout the soybean genome. For example, Chr 19 contained the most *GmPME* genes (13 genes), whereas merely a single gene located on Chr 11, Chr 12, Chr 18 and Chr 20, respectively. Moreover, the amount of *GmPME* genes on soybean chromosomes did not exhibit correlations to the length of chromosomes. To better reveal the distributing tendency of *GmPME* genes, a series of gradient colors were endowed on soybean chromosomes. And the gradient colors were depicted based on the gene densities on soybean chromosomes, which were deduced from the gene numbers in the 300-kb genetic intervals on different soybean chromosomes (Additional file 5: Table S5). Interestingly, most identified *GmPME* genes tended to gather in regions with high gene densities.

### Duplication, synteny and evolution analyses of *GmPME* gene members

**Fig. 5:**
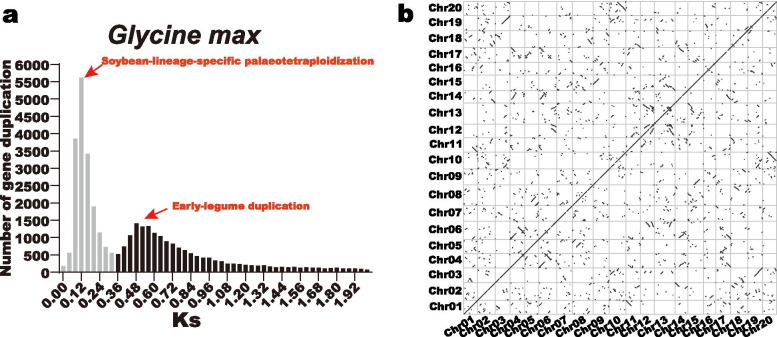
Frequency histogram of synonymous substitutions (Ks) of soybean homologous gene pairs and genome duplicated gene dotplot. **a** Frequency histogram of synonymous substitutions (Ks) of soybean homologous gene pairs. The two peaks were corresponding to soybean-lineage-specific palaeotetraploidization (in gray) and early-legume duplication (in black), respectively. **b** Genome duplicated gene dotplot. The tandemly and segmentally duplicated gene pairs were identified and depicted in this plot with dots. Tandemly duplicated gene pairs were located at the diagonal line and the rest dots represented the segmentally duplicated gene pairs

Gene duplications are regarded as one of the major driving forces that promote genome evolutions [32]. Soybean is a palaeopolyploid, which under went two whole genome duplication (WGD) events near 59 and 13 million years ago, integrating a highly duplicated genome that most genes displayed multiple copies [33]. In this study, we calculated the synonymous substitutions (Ks) of soybean homologous gene pairs throughout the soybean genome (Additional file 6: Table S6), and the Ks frequency distribution histogram was depicted in Fig. 5a. As is shown in Fig. 5a, the distribution of Ks presented two peaks, corresponding to the recent soybean-lineage-specific palaeotetraploidization event (in gray color) and the early-legume duplication event (in black color), respectively.

**Fig. 6:**
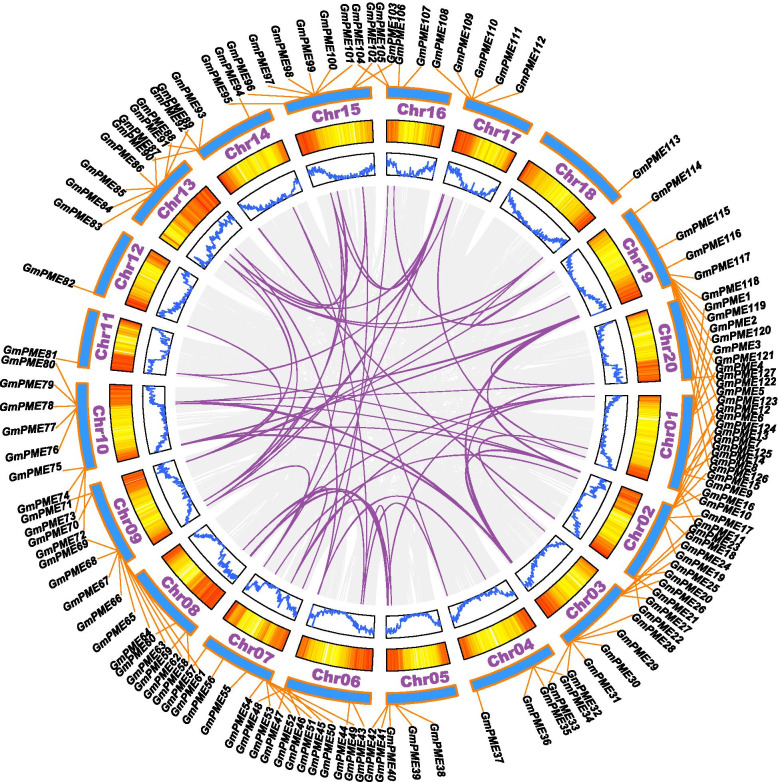
Circos plot representing the collinearity of soybean homologous genes. Genome-wide collinear blocks were set as the background in gray and the duplicated *GmPME* gene pairs were linked and highlighted with purple curves. In addition, each soybean chromosome was attached with 300-kb gene density information depicted by heatmap and wave graph

Early research demonstrated that the expansions of plant gene families were mainly promoted by segmental and tandem duplication events [34, 35]. Segmental duplications often happened during polyploidization events with chromosome rearrangements, resulting in a large amount of duplicated chromosomal blocks in the genomes [36]. By contrast, tandem duplications were defined as the events that multiple adjacent homologous gene family members (two or more members) cluster on a single chromosome [37]. To gain a better overview of the two major gene duplication events in soybean, we explored and filtered the segmentally and tandemly duplicated gene pairs throughout the soybean genome (Additional file 7: Table S7 and Additional file 8: Table S8). And the duplicated gene pairs were further depicted with a series of dots in the soybean genome duplicated gene dotplot (Fig. 5b). According to the definitions of segmental and tandem duplications, the dots distributed at the diagonal line were corresponding to the tandem duplicated gene pairs, and the rest dots were the segmentally duplicated gene pairs. Our results support that soybean presents a highly duplicated genome, which is abundant in  segmental and tandem duplicated gene pairs. In the *GmPME* gene family, we detected 18 tandemly duplicated gene pairs (Additional file 9: Table S9), which were linked by red arcs throughout 11 soybean chromosomes in Fig. 4. In contrast, we found 90 segmentally duplicated gene pairs (Additional file 9: Table S9), which were five times of tandemly duplicated ones and jointed by purple curves in collinear Circos plot in Fig. 6. Taken together, we speculated that the expansion of the *GmPME* gene family was both associated with tandem and segmental duplication events, whereas segmental duplications took the lead in deriving new *GmPME* gene members.

**Fig. 7:**
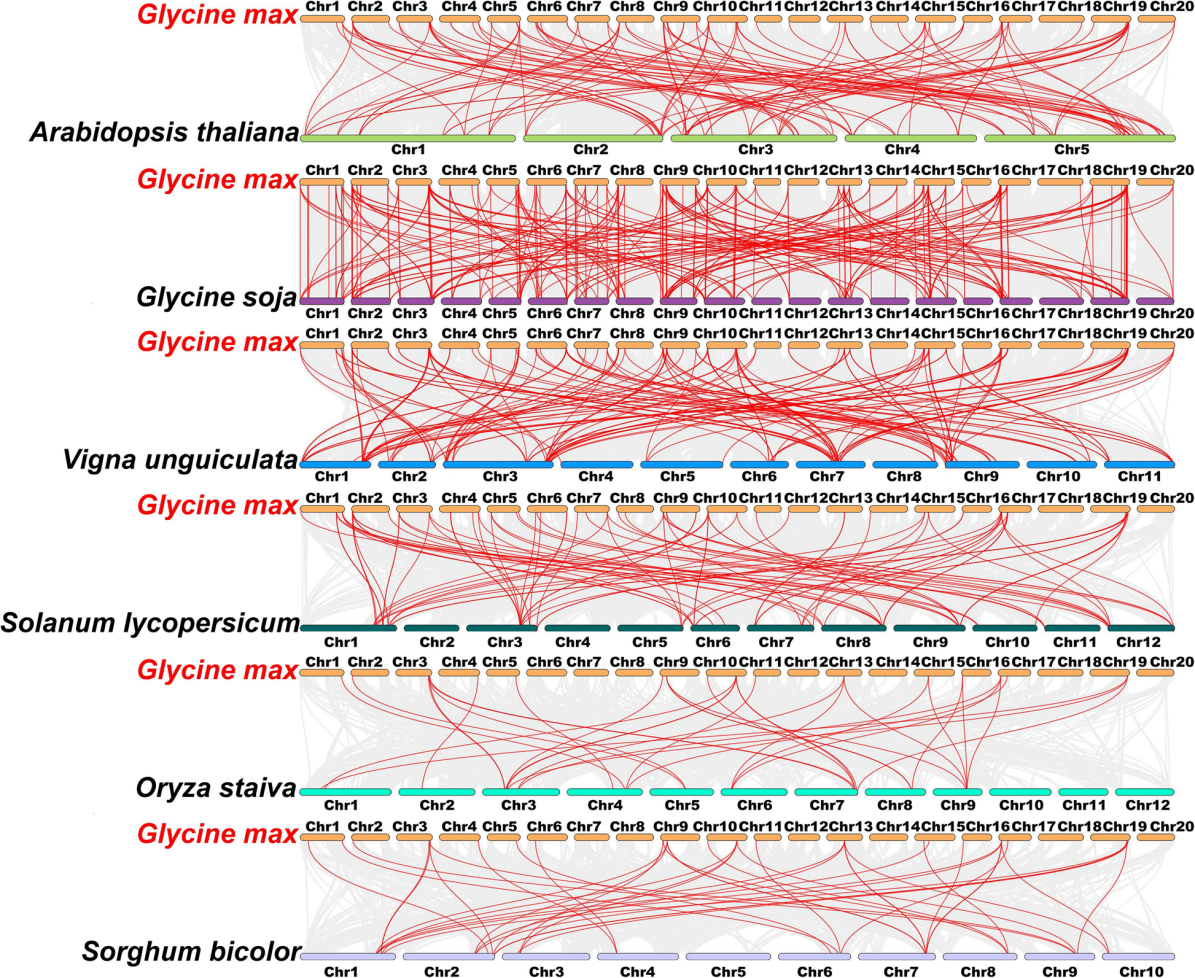
Schematic displaying the synteny of PME members between soybean and six representative species. The syntenic blocks were set as the gray background, and the syntenic PME members were highlighted with red curves

**Fig. 8:**
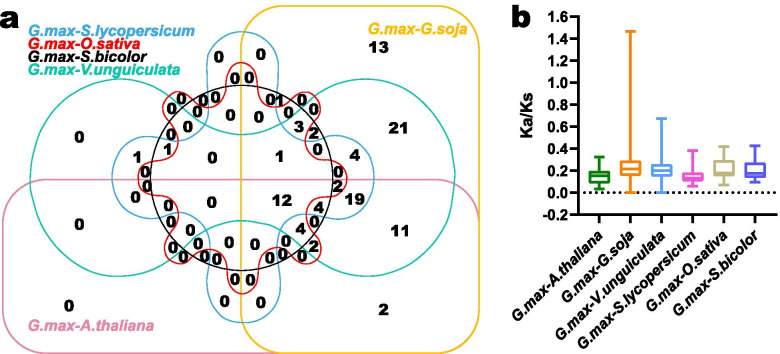
Venn diagram representing non-redundant syntenic PME members in soybean and other species, and evolutionary constraint calculations of orthologous *PME* genes. **a** Venn diagram representing non-redundant syntenic PME members in soybean and other species. **b** Box plot for the ratios of nonsynonymous to synonymous substitutions (Ka/Ks) in orthologous *PME* genes. And ‘*G. max*’, ‘*A. thaliana*’, ‘*G. soja*’, ‘*V. unguiculata*’, ‘*S. lycopersicum*’, ‘*O. sativa*’ and ‘*S. bicolor*’ were *Glycine max*, *Arabidopsis thaliana*, *Glycine soja*, *Vigna unguiculate*, *Solanum lycopersicum*, *Oryza sativa* and *Sorghum bicolor*, respectively

To seek the syntenic relations between *GmPME* members and the *PME* gene members in different species, we recruited four dicots (*Arabidopsis thaliana*, *Glycine soja*, *Vigna unguiculata* and *Solanum lycopersicum*) and two monocots (*Oryza sativa* and *Sorghum bicolor*), and carried out the synteny analyses. Consequently, 255, 149, 87, 65, 48, 38 and 33 *GmPME* orthologous genes were identified in *Glycine soja*, *Vigna unguiculata*, *Solanum lycopersicum*, *Arabidopsis thaliana*, *Sorghum bicolor* and *Oryza sativa*, respectively (Additional file 10: Table S10). Furthermore, we depicted six comparative syntenic graphs representing the orthologous gene pairs in soybean and the recruited species, and the *PME* orthologous gene pairs were linked by red curves (Fig. 7). Besides, we filtered the non-redundant *GmPME* genes that exhibited the syntenic relationships among soybean and the other six species (Additional file 11: Table S11), and an interactive Venn diagram of the non-redundant *GmPME* genes throughout the different species was displayed in Fig. 8a. Totally 12 *GmPME* genes contained the corresponding orthologous genes in all recruited species (Fig. 8a), which were further emphasized in bold in Table S11 (Additional file 11). The conjoint orthologous gene pairs throughout distinct species may be useful for conducting relevant evolutionary studies of *GmPME* genes. Importantly, the evolutionary constraints that acted on *GmPME* genes and their orthologous genes in the six species were also assessed by calculating the Ka/Ks (non-synonymous substitution/synonymous substitution) ratios (Additional file 10: Table S10). And all the acquired Ka/Ks ratio values were used to draw a box plot in Fig. 8b. It is noteworthy that most *GmPME* orthologous gene pairs showed Ka/Ks < 1, which indicated that the *GmPME* gene family may experience strong purifying selective pressures during evolution [38].

### *Cis*-elements in the promoters of *GmPME* genes

**Fig. 9:**
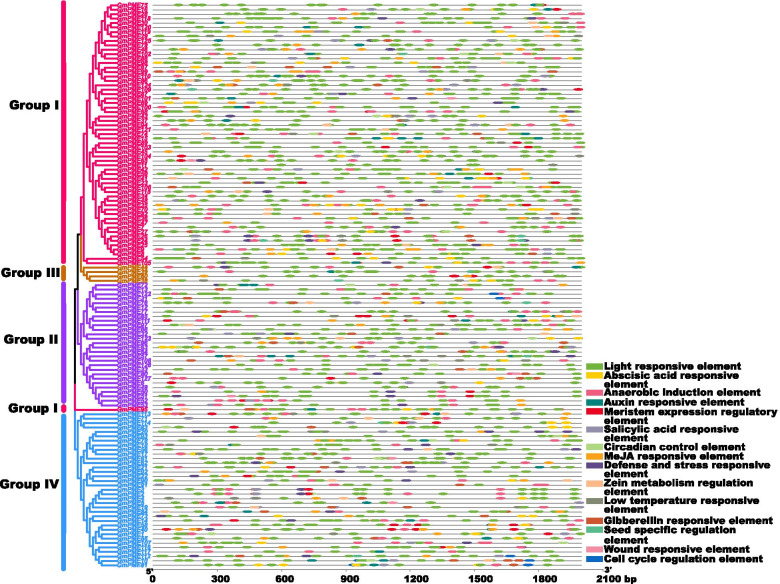
Schematic of *cis*-element patterns in the upstream 2000-bp genetic block of *GmPME* genes

To explore the potential roles of *GmPME* gene members participating in various soybean biological processes, we extracted the promoter region sequences of *GmPME* genes (the 2000 bp upstream sequences from gene initiation codons) and carried out the *cis*-element analyses (Additional file 12: Table S12). Furthermore, the *cis*-elements in gene promoter regions were proportionally illustrated (Fig. 9). As is illustrated in Fig. 9, a total of 15 distinct types of *cis*-elements were displayed. Importantly, *cis*-elements including the abscisic acid responsive element, the auxin responsive element, the salicylic acid responsive element, the MeJA responsive element, the gibberellin responsive element, the defense and stress responsive element and the low temperature responsive element were broadly existed, which suggested the *GmPME* gene members may link to the responses of distinct hormones and stresses. Besides, some plant growth and development associated *cis*-elements like the light responsive element, the anaerobic induction element, the meristem expression regulatory element, the circadian control element and the cell cycle regulation element were also detected. To sum up, the *GmPME* genes may take crucial roles in soybean growth and development as well as in various hormones and stress responses.

### Expression patterns of *GmPME* genes in distinct tissues

**Fig. 10:**
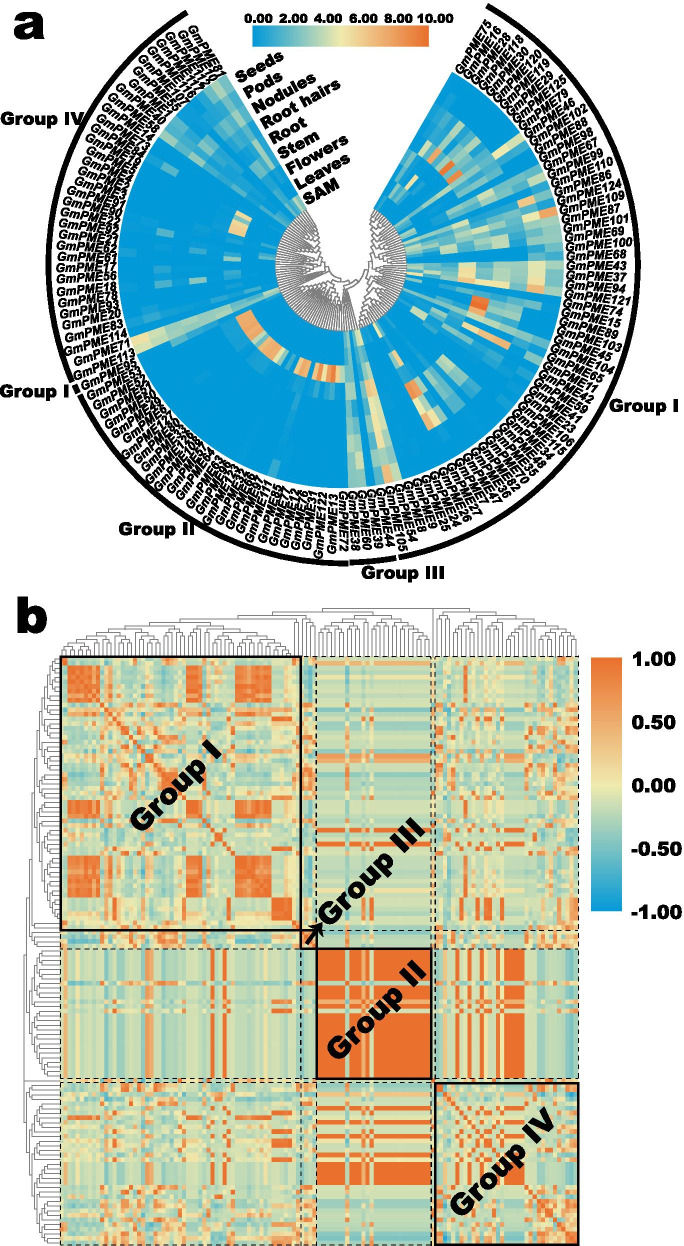
Expression profiling of *GmPME* genes in multiple tissues on Phytozome. **a** Expression profiles of *GmPME* genes in multiple tissues on Phytozome. The extracted FPKM values were Log_2_ normalized to display the heatmap. SAM: Shoot apical meristem. **b** Gene expression correlation heatmap of the *GmPME* gene in diverse tissues. Red: positively correlated; blue: negatively correlated

The expression profiles of the 127 identified *GmPME* genes in various tissues, including shoot apical meristem (SAM), leaves, flowers, stem, root, root hairs, nodules, pods and seeds were extracted from Phytozome in Table S13 (Additional file 13). Then the extracted data were Log_2_ normalized to generate a heatmap in Fig. 10a. In general, the *GmPME* genes in different hereditary groups or clades showed distinct expression patterns. For instance, most *GmPME* genes in the Group II clade displayed superior expression levels in flowers compared to other tissues. In comparison, some *GmPME* gene members in the Group I clade exhibited high expression levels in other tissues like root, nodules, pods and seeds. And the most *GmPME* genes in the Group III and Group IV clades were at relatively low expression levels throughout tissues.

To further explore the expression patterns of the *GmPME* genes in various tissues, we calculated the gene expression correlation coefficients among the identified *GmPME* gene members in Table S14 (Additional file 14). Based on the acquired correlation coefficients, a phylogenetically clustered heatmap was drawn in Fig. 10b. According to the different clades, the heatmap was separated into different blocks, and diverse groups were enclosed by solid boxes and labeled with their names in bold. As is shown in Fig. 10b, most *GmPME* gene members in the Group II and Group III clades showed positive correlations with the internal members and exhibited similar expression patterns throughout the clades. By contrast, in the Group I and Group IV clades, most *GmPME* gene members displayed divergent expression patterns throughout the internal or external clades.

**Fig. 11:**
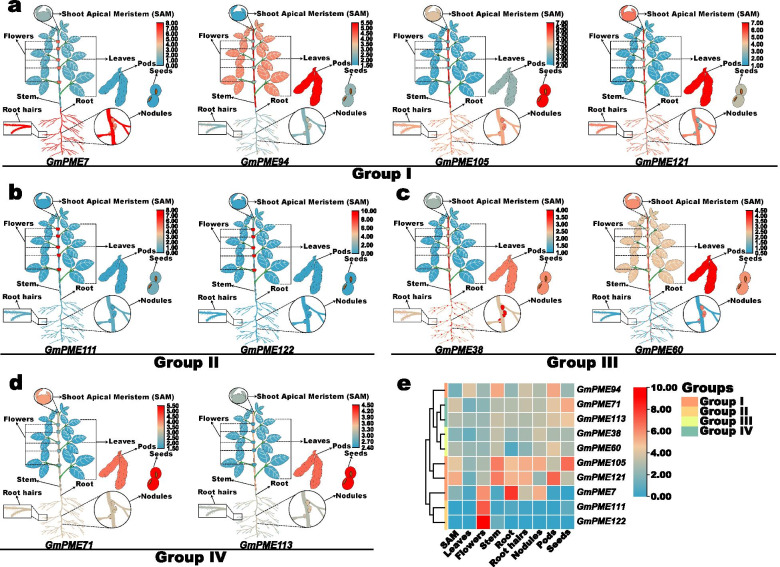
A series of fancy heatmaps and integrative grouped heatmap exhibiting the different expressed representative *GmPME* genes. **a** Fancy heatmaps of different expressed representative *GmPME* genes in the Group I clade. **b** Fancy heatmaps of different expressed representative *GmPME* genes in the Group II clade. **c** Fancy heatmaps of different expressed representative *GmPME* genes in the Group III clade. **d** Fancy heatmaps of different expressed representative *GmPME* genes in the Group IV clade. **e** The integrative grouped heatmap exhibits the different expressed representative *GmPME* genes

To better visualize the diverse expression patterns of *GmPME* genes in tissues, we concomitantly selected ten representative *GmPME* genes and created fancy heatmaps representing relevant gene expressions (Fig. 11). As is shown in Fig. 11a, four *GmPME* genes were from the Group I clade. *GmPME7* mainly expressed in flowers, root and nodules; *GmPME94* showed relatively high expressions in leaves, stem and pods; *GmPME105* highly expressed in stem, root, root hairs, nodules and seeds; *GmPME121* displayed superior expressions in SAM, stem, root, root hairs and pods. Besides, *GmPME111* and *GmPME122* were from the Group II clade and uniformly exhibited high expressions in flowers (Fig. 11b). *GmPME38* and *GmPME60* from the Group III clade were depicted in Fig. 11c. *GmPME38* presented relatively high expression levels in stem, root hairs, nodules, pods and seeds. Whereas, *GmPME60* displayed considerable expressions in SAM, stem, nodules, pods and seeds. The two extracted genes (*GmPME71* and *GmPME113*) from the Group IV clade exhibited superior expressions in pods and seeds, and *GmPME71* simultaneously owned relatively high expression in SAM (Fig. 11d). Moreover, the expression data of the ten extracted genes were further together normalized and integrated to acquire a heatmap to globally display the expression patterns of the representative genes in distinct clades (Fig. 11e). Overall, our findings supported that the *GmPME* genes diversely expressed in tissues, which may more or less highlight the important regulating roles of the key *GmPME* genes during soybean plant development.

### Expression profiling of *GmPME* genes in flower buds of soybean cytoplasmic male sterile line NJCMS1A and its maintainer NJCMS1B

**Fig. 12:**
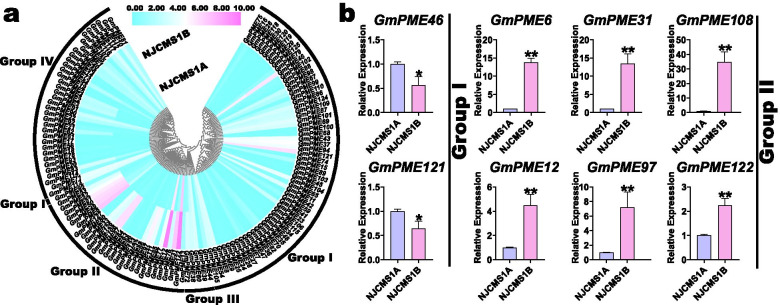
Expression profiling of *GmPME* genes in soybean flower buds from cytoplasmic male sterile line NJCMS1A and its maintainer NJCMS1B. **a** Expression profiles of *GmPME* genes in soybean flower buds from NJCMS1A and NJCMS1B. **b** The quantitative RT-PCR analyses of eight selected representative *GmPME* genes from the Group I and Group II clades. The results of quantitative RT-PCR were normalized to the *GmActin* housekeeping gene. The error bars indicated the standard deviations and the values in plots were corresponding to the mean ± standard deviation (SD) of three independent biological replicates. Asterisks demonstrated that the selected *GmPME* genes were significantly up- or down-regulated compared with those in soybean flower buds from NJCMS1A (* *P* < 0.05, ** *P* < 0.01, Student’s *t*-test)

The utilization of crop plant heterosis is a potential way to fulfill yield breakthroughs in the future [39]. In this study, we extracted the expression profiles of the identified *GmPME* genes in the flower buds of soybean cytoplasmic male sterile line NJCMS1A and its maintainer NJCMS1B from the published transcriptome data (Additional file 15: Table S15) [40]. Moreover, the gene expression data was Log_2_ normalized and acquired a phylogenetically clustered heatmap (Fig. 12a). As is shown in the figure, the expressions of the *GmPME* genes in the maintainer NJCMS1B are universally higher than those in cytoplasmic male sterile line NJCMS1A. In the Group I and Group II clades, some *GmPME* genes, for examples, *GmPME46* and *GmPME121* in the Group I clade as well as *GmPME12* and *GmPME122* in the Group II clade, were at relatively high expression levels both in NJCMS1A and NJCMS1B. Notably, the most highly expressed *GmPME* genes were in NJCMS1B and gathered in the Group II clade. Whereas, in the Group III and Group IV clades, the *GmPME* genes were universally low expressed.

Concomitantly, the gene expression data was row-scaled with the zero-to-one method to further exhibit the expression differences of *GmPME* genes in cytoplasmic male sterile line NJCMS1A and its maintainer NJCMS1B (Additional file 16: Fig. S1). Importantly, in the Group I and Group II clades, we respectively selected two and six representative *GmPME* genes, which were at high expression levels and differently expressed in NJCMS1A and NJCMS1B (Additional file 15: Table S15), and carried out the quantitative RT-PCR analyses (Fig. 12b). As a result, all the selected genes showed significantly distinct transcript levels in NJCMS1A and NJCMS1B (* *P* < 0.05 or ** *P* < 0.01, Student’s *t*-test). For instance, *GmPME46* and *GmPME121* in the Group I clade showed superior expressions in NJCMS1A than those in NJCMS1B (* *P* < 0.05). By contrast, the six representative genes in the Group II clade exhibited significantly higher transcription levels in NJCMS1B than those in NJCMS1A (** *P* < 0.01). Collectively, the diverse expressions of the identified *GmPME* genes, especially for the representative ones, may directly or indirectly manifest their important roles in regulating flower bud development in soybean cytoplasmic male sterile line NJCMS1A and its maintainer NJCMS1B.

### Expression patterns of *GmPME* genes during soybean seed germination

**Fig. 13:**
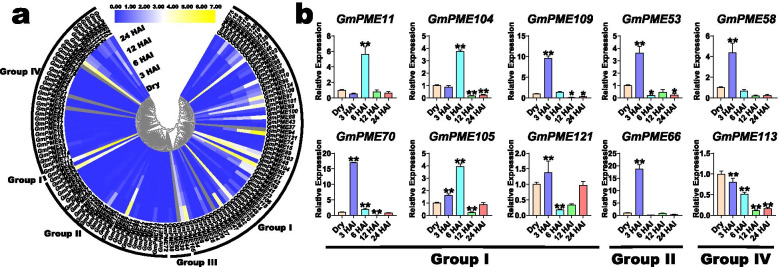
Expression profiling of *GmPME* genes in soybean embryonic axes during seed germination. **a** Expression profiles of *GmPME* genes in soybean embryonic axes during seed germination. HAI: hours after imbibition. *GmPME* genes that lacked expression information in the transcriptome data were depicted with the gray color in the heatmap. **b** The quantitative RT-PCR analyses of ten selected representative *GmPME* genes from the Group I, Group II and Group IV clades. The results of quantitative RT-PCR were normalized to the *GmActin* housekeeping gene. The error bars indicated the standard deviations and the values in plots were corresponding to the mean ± standard deviation (SD) of three independent biological replicates. Asterisks demonstrated that the selected *GmPME* genes were significantly up- or down-regulated compared with those in the dry soybean embryonic axes (* *P* < 0.05, ** *P* < 0.01, Student’s *t*-test)

Previous studies demonstrated that the PME activity may contribute to the temporal regulations of mechanical properties of the cell walls, and thereby affect seed germination [11, 41]. In the current investigation, the expression data of the identified *GmPME* genes in the embryonic axes during soybean seed germination was derived from the transcriptome data (Additional file 17: Table S16) [42]. As a result, the expression profiles of 121 *GmPME* genes were acquired, and the extracted data were Log_2_ normalized to draw a phylogenetically clustered heatmap (Fig. 13a). And five time points (dry, 3 HAI (hours after imbibition), 6 HAI, 12 HAI and 24 HAI) were investigated during soybean seed germination. Overall, most *GmPME* genes showed relatively low expression levels during soybean germination. Whereas, some *GmPME* genes were dramatically up-regulated over time throughout different clades, which may indicate these genes participate in the regulations of soybean seed germination.

Likewise, the gene expression heatmap was additionally row-scaled with the zero-to-one method to show the expression variations of each *GmPME* gene during soybean seed germination processes (Additional file 18: Fig. S2). To further confirm whether the *GmPME* genes regulate the soybean seed germination processes, ten representative *GmPME* genes, whose expression levels were relatively high at diverse time points, were carefully selected from different genetic clades (Group I, Group II and Group IV) and carried out the quantitative RT-PCR validations (Fig. 13b). As a whole, most selected *GmPME* genes (except for *GmPME113*) were remarkably upregulated at the very beginning during soybean seed germination (3 HAI or 6 HAI). Besides, the selected genes exhibited various expression patterns, which may indicate they regulate seed germination in different patterns (Fig. 13b).

## Discussion

Pectin methylesterases (PMEs) contribute to the balance of solidifying and softening of plant cell walls, which are crucial for plant growth and diverse biological processes [43, 44]. The rapid development of whole-genome sequencing technologies boosted up the recent identifications and analyses of *PME* gene families in different plants. Despite the soybean (*Glycine max*) genome having been well sequenced, however, there were few reports on *Glycine max PME* (*GmPME*) genes. In the current study, we comprehensively explored and characterized the *PME* gene family in soybean.

Totally 127 *GmPME* genes were identified from the soybean *Wm82.a2.v1* genome and were later divided into four hereditary clades (Fig. 1 and Additional file 1: Table S1). Soybean underwent two whole genome duplication events (Fig. 5a) and owned a highly duplicated genome coupled with multiple gene copies. The number of *PME* genes are varied in species. Here, we acquired more *PME* gene members in soybean than those reported in flax (105 members) [45], Arabidopsis (66 members) [18], rice (59 members) [19], strawberry (54 members) [2] and maize (43 members) [20]. Plant genome evolutions often along with segmental and tandem duplications that resulted in the expansions of different gene families [46]. As is depicted in Figs. 4 and 6, segmental duplications mainly drove the expansion of *GmPME* genes, and this was also following the results in the soybean genome  duplication dotplot (Fig. 5b). Interestingly, the identified *GmPME* genes are unevenly distributed on 20 soybean chromosomes, which might be caused by gene replications or partially fragmental gene duplications of the genome during soybean evolution [21, 47].

Introns were reported to be associated with the expansions of gene families during plant evolutions, which usually raised at the earlier stages of gene family expansion and gradually wiped over time [17, 48, 49]. Besides, the enlargement of introns was regarded as an effective way to gain new gene functions [50]. By investigating the gene structures, we noticed that introns broadly existed in *GmPME* genes (Fig. 2). For instance, *GmPME54*, *GmPME105*, *GmPME106* and *GmPME11*5 in the Group I clade; *GmPME13* and *GmPME96* in the Group II clade; *GmPME114* in the Group IV clade contained large-spanning introns, which may indicate that they were relatively novel *GmPME* genes. In general, *GmPME* genes that clustered in the same clade exhibited analogous exon-intron patterns and conserved domain components (Fig. 2), and this may demonstrate that they conduct analogical gene functions. Notably, most *GmPME* gene members in the Group I, II and III clades also have PMEI conserved domains at the N-terminuses (Fig. 2), which were similar to the *PME* gene members reported in cotton, maize and strawberry [2, 20, 21]. Compared to the C-terminuses, the N-terminuses displayed relatively variable characteristics during evolution processes. *GmPME* gene members that contained both PME and PMEI conserved domains may cause structural as well as functional alterations of PMEs. And to some extent, the conservation of PMEI domains also facilitates keeping the fundamental functions of the gene family, improves the gene diversities, and shrinks the selection pressures during evolution [21].

To further explore the hereditary mechanisms of *GmPME* genes, we carried out synteny analyses of *PME* gene members between soybean and six representative species (four dicots and two monocots) in Fig. 7. Compared to those in monocots, *PME* genes in dicots presented superior syntenic relations with *GmPME* genes. And *Glycine soja* and *Glycine max* (soybean) exhibited the best synteny, which highlighted the evolutionary differentiation in distinct species [17]. In this study, evolutionary constraints acting on the *GmPME* gene family were evaluated by calculating the Ka/Ks ratios of the orthologous *PME* gene pairs. Despite most ratios showing Ka/Ks < 1, whereas, we also found that some orthologous gene pairs between *Glycine soja* and *Glycine max* exhibited Ka/Ks > 1 (Additional file 10: Table S10), which manifested these orthologous *PME* gene pairs underwent positive selections [51].

The *cis*-element analyses in the current study further supported the potential roles of the identified *GmPME* genes in regulating soybean developments and responses to various biological processes (Fig. 9). By exploring the expression patterns of *GmPME* genes in different tissues, we acquired a diverse transcript abundance (Fig. 10) and manifested that they might dissent in gene functions. Besides, the homologous *GmPME* genes in the specific clade may have similar functions. Remarkably, most *GmPME* gene members in the Group II clade showed alike expression patterns (Fig. 10b), and they tended to show high gene expression levels in the flower buds in NJCMS1B (Fig. 12a). In Arabidopsis, *PME* genes in the group 2 clade were highly or uniquely expressed in the flower buds and were proved to be involved in pollen maturation and pollen tube growth [18]. In cotton, transcriptome analysis revealed that most *PME* genes expressed higher in flower buds of the fertile individuals than those in the CMS-D8 line [52]. Moreover, semi-qRT-PCR showed that 13 maize *PME/PMEI* genes were differentially expressed in the anthers of CMS fertile and sterile plants [20]. The utilization of heterosis with the CMS systems is a feasible way to increase soybean yield [29, 30, 40]. In this study, we conducted gene expressing dissection between soybean cytoplasmic male sterile line NJCMS1A and its maintainer NJCMS1B by investigating previous transcriptome data (Fig. 12a and Additional file 15: Table S15) [40]. Similar to the findings in other plant species, most *GmPME* genes exhibited higher transcript levels in NJCMS1B than those in male sterile line NJCMS1A, and most high expression genes were gathered in the Group II clade [20, 52]. Importantly, distinct representative *GmPME* from the Group I and II clades were selected and conducted the quantitative RT-PCR analyses. As a whole, the expression patterns of selected *GmPME* genes displayed by quantitative RT-PCR matched the transcriptome data, which further supported the validity of the early transcriptome analysis [40].

Seed germination is one of the principal processes in the plant life cycle [53]. Seeds with higher vigor and quality guaranteed crop yield [17]. PMEs play an important role in plant developments, including the regulations of seed germination [2, 12]. A previous study in yellow cedar demonstrated that PME activity positively correlated with seed germination performance [54]. In Arabidopsis, PME activities rarised until the completed time of testa rupture, then gradually decreased with the weakening and rupture of endosperm [11]. Besides, the pectin-modifying enzyme genes exhibited high transcript levels during the first 24 h of Arabidopsis seed germination [12, 55]. In the current research, we explored the expression patterns of *GmPME* genes in soybean embryonic axes during seed germination [42]. Notably, different *GmPME* genes displayed diverse expression patterns throughout clades, which highlighted their distinct roles in regulating soybean seed germination (Fig. 13a). Simultaneously, we selected ten representative *GmPME* genes from different clades and conducted the quantitative RT-PCR analyses. In general, the expressions of most selected *GmPME* genes, except for *GmPME113*, were remarkably up-regulated at early time points of soybean germination than dramatically down-regulated over time, which highlighted the characteristics of the selected *GmPME* genes in temporal regulation during seed germination. It is worth noting that the transcript levels of the selected *GmPME* genes in the quantitative RT-PCR presented divergences compared with those expression patterns displayed in transcriptome data (Fig. 13a and Additional file 17: Table S16) [42]. For example, *GmPME105*, *GmPME109* and *GmPME121* in the Group I clade, as well as *GmPME66* in the Group II, showed earlier gene up-regulations compared to those in the transcriptome data (Fig. 13a), which may produce by the seed soaking step in this study that boosted relevant gene up-regulation in advance [17]. Besides, the rest selected *GmPME* also displayed more or less differences with the transcriptome data. The possible cause may be due to soybean varieties used in the previous study (cv. ‘BRS 284’) and the present research (cv. ‘Williams 82’) exist hereditary disparities [42]. Taken together, we speculated that relevant *GmPME* genes may take possible roles in modulating soybean seed germination.

In summary, we conducted a comprehensive survey on *GmPME* genes and the findings in the current study may provide reference points in future gene function studies. In this study, we demonstrated the potential roles of the identified *GmPME* genes during flower bud developments and seed germination by carrying out gene expression profiling. Nevertheless, the *GmPME* genes still need to be endowed with further functional investigations to illustrate their detailed roles in diverse biological courses.

## Conclusions

The *PME* gene families have been examined in several plant species and related to various important developmental and biological processes. In comparison, associated research about the soybean *PME* gene family is still limited. This study provided a comprehensive identification and characterization of the *GmPME* genes, including gene structures and conserved domain properties, gene chromosomal location depictions, gene family member phylogeny and evolutionary investigations, *cis*-element exhibitions in the gene promoter regions, gene expression patterns in different tissues, gene expression profiling during flower bud development as well as seed germination processes. In conclusion, our work could pave the way for future functional study of *PME* genes in soybean, especially served valuable references for the regulations of *PME* genes during soybean flower bud development and seed germination.

## Methods

### Identification of *PME* genes in soybean

Gene identification was according to the method of Wang et al. [17]. Soybean genome (the *Wm82.a2.v1* version) and its annotation file were obtained from Phytozome (https://phytozome-next.jgi.doe.gov/). All the reported AtPME protein sequences were downloaded from TAIR (https://www.arabidopsis.org/) and set as the query sequences to extract the most representative GmPME protein sequences by TBtools software [56]. Moreover, the extracted GmPME proteins were further checked by NCBI BLASTp (https://blast.ncbi.nlm.nih.gov/Blast.cgi?PROGRAM=blastp&PAGE_TYPE=BlastSearch&LINK_LOC=blasthome). The conserved domains of GmPME proteins were analyzed by SMART (http://smart.embl.de/). Proteins less than 100 aa (amino acid), without the PME domains or with obvious errors were manually filtered. The ExPASy online tool (http://expasy.org/tools/) was adopted for examining the molecular weight (MW) and the isoelectric point (pI) of the GmPME proteins, and CELLO (http://expasy.org/tools/) were recruited for *GmPME* gene subcellular localization predictions.

### Phylogenetics and classifications of GmPME members

The phylogenetic tree was deduced by MEGA 7.0 (https://www.megasoftware.net/) with the neighbor-joining (NJ) method [57]. The AtPME and GmPME protein sequences were aligned by using the MUSCLE approach [58]. The phylogenetic tree was constructed with the Poisson model, pairwise deletion, and 1000 bootstrap replications. The GmPME members were divided into distinct groups based on the classifications of AtPME members in Arabidopsis. Both FigTree (http://tree.bio.ed.ac.uk/software/figtree/) and Adobe Illustrator (https://www.adobe.com/products/illustrator/free-trial-download.html) were recruited to decorate the original phylogenetic tree.

### Gene structure and motif pattern illustrations

Based on the soybean genome annotation file and the acquired SMART conserved domain information, we displayed the *GmPME* gene structures by TBtools [56]. For motif patterns, the GmPME proteins were submitted and carried out the conserved motif scanning on the MEME website (http://meme-suite.org/tools/meme) by setting the MEME-motif as ten. And the obtained MEME-motifs were ulteriorly matched to the conserved domains (Additional file 4: Table S4). Besides, the Seq Logos of the MEME-motifs were illustrated by TBtools. Adobe Illustrator software was adopted to polish the graphs.

### Chromosomal location, duplication, synteny and evolution analyses of *GmPME* gene family

According to the soybean genome annotation file, we extracted the 300-kb hereditary interval gene densities (Additional file 5: Table S5) and further transformed them into the gradient colored heatmap on each soybean chromosome. The chromosomal locations of *GmPME* genes were depicted with TBtools based on the soybean genome annotation file [56]. Gene family duplication analysis was carried out by calculating the synonymous substitutions (Ks) of the homologous gene pairs with TBtools. The Ks frequency distribution histogram was used to evaluate soybean whole genome duplication (WGD) events. Soybean whole genome segmentally and tandemly duplicated gene pairs were detected and further depicted in the genome duplicated genes plot by TBtools. For *GmPME* segmentally and tandemly duplicated homologous gene pairs, we linked them by the red arcs in the gene chromosomal location plot and the purple curves in the collinear Circos plot, respectively [59]. Besides, synteny analyses among the *PME* gene members of soybean and other species on Phytozome including *Arabidopsis thaliana* (TAIR annotation release 10), *Glycine soja* (V1.1), *Vigna unguiculata* (V1.1), *Solanum lycopersicum* (ITAG3.2), *Oryza sativa* (MSU annotation release 7.0) and *Sorghum bicolor* (V3.1.1) were also explored. And syntenic graphs of multiple species were generated by TBtools. Moreover, *GmPME* genes that contained orthologous genes in distinct species were summarized by the Venn diagram. The ratios of nonsynonymous substitution (Ka) to synonymous substitution (Ks) of *GmPME* orthologous gene pairs were computed with TBtools, and the acquired results were presented by box plot with Graphpad Prism 8 (https://www.graphpad.com/scientific-software/prism/). The derived graphs were further edited by Adobe Illustrator software.

### *Cis*-element analyses of *GmPME* gene promoter regions

Based on the extractions of the *GmPME* gene upstream 2000 bp sequences with TBtools, the *cis*-elements in gene promoter regions were explored with PlantCARE online tools (http://bioinformatics.psb.ugent.be/webtools/plantcare/html/) [60]. The schematic of *cis*-element distributions in *GmPME* gene promoter regions was displayed by TBtools. Adobe Illustrator was used to modifying the graph.

### Expression profiling of *GmPME* genes

The expression profiles of *GmPME* genes in various tissues were extracted from transcriptome data on Phytozome. Additionally, the expression correlation matrix of *GmPME* genes in tissues was calculated by Omicshare online tools (https://www.omicshare.com/tools/Home/Soft/getsoft). And the acquired matrix was adopted to generate the correlation heatmap by TBtools [56]. To better exhibit gene expression patterns in tissues, distinct representative *GmPME* genes were selected from different hereditary clades to draw a panel of gene expression fancy heatmap by using TBtools. Besides, the transcriptional levels of the identified *GmPME* genes in flower buds of soybean cytoplasmic male sterile line NJCMS1A and its maintainer NJCMS1B as well as the expression patterns of the *GmPME* genes in soybean embryonic axes during seed germination were investigated from early research data [40, 42]. All the acquired transcriptome data were assessed by the FPKM (fragments per kilobase million) values. And the FPKM values were Log_2_ normalized, then using TBtools to depict associated gene expression heatmaps. Adobe Illustrator was used to modifying the graph.

### Plant materials and samplings

Soybean cytoplasmic male-sterile line NJCMS1A was derived from the consecutive backcross approaches with the donor parent N8855 and the recurrent parent N2899 (further designated as NJCMS1B) cultivars [61]. And cultivar Williams 82 was adopted for seed germination assay. All plant materials were planted and harvested in 2019 at Dangtu Experimental Station, National Center for Soybean Improvement, Nanjing Agricultural University, Dangtu, Anhui, China. Samplings were originated from three independent individuals of the plant materials. During flowering growth stages, different size flower buds of NJCMS1A and NJCMS1B were collected and frozen in liquid nitrogen and then stored at − 80 °C [29]. According to Wang et al., 40 intact soybean seeds for germination assay were selected from the independent plants and dried for 3 days at 40 °C to keep uniform moisture [17]. And the selected seeds were pre-disinfected by 0.05% potassium permanganate solution for 5 min and washed with deionized water. Seed germination was performed by using the germination pouches (PhenoTrait Technology Co., Ltd.) followed by the instruction book: the disinfected seeds were soaked in deionized water for 2 h, then placed the pouches with seeds on the germination pouch shelf in a temperature-controlled incubator at 25 °C in dark. Five time points during seed germination including dry, 3 HAI (hours after imbibition), 6 HAI and 12 HAI and 24 HAI were investigated in the current study. For each time point, 20 healthy embryonic axes were selected and separated from the testing seed for RNA extractions [17].

### RNA extraction and quantitative RT-PCR validation

Three independent plants consisting of three biological replicates were adopted for RNA extractions and quantitative RT-PCR validations. Total RNA was isolated from the samples by using the RNAprep pure plant kit (TIANGEN, Beijing, China) [17]. And the quality of extracted RNA was examined by electrophoresis and then quantified with a Nanodrop ND-1000 spectrophotometer (Nanodrop, Wilmington, DE, USA) [17]. We selected 17 representative *GmPME* genes and conducted the quantitative RT-PCR validations to ulteriorly explore the expression patterns of *GmPME* genes during soybean flower bud developments as well as seed germination. The specific quantitative RT-PCR primers were designed by Primer Premier 5 and listed in Table S17 (Additional file 19). The removement of genomic DNA and conversion of RNA to cDNA were conducted by using the HiScript II 1st Strand cDNA Synthesis Kit (Vazyme Biotech, Nanjing, China) [17]. A BioRad CFX96 real-time system was adopted to perform the quantitative RT-PCR assay with SYBR qPCR Master Mix (Vazyme Biotech, Nanjing, China) [17]. Triplicate quantitative assays were performed on each cDNA sample and analyzed by a 2^−△△CT^ method by setting the housekeeping *GmActin* gene as the internal control [62].

### Statistical analyses

Student’s *t*-test was examined by Graphpad Prism 8 and the *P*-value cut-off of 0.05 was the criterion to determine whether the test was significantly different or not. Error bars in the column diagrams were standard deviations (SD) from the independent biological replicates.

## Supplementary Information

**Additional file 1: Table S1.** The 127 identified *GmPME* genes in this study.

**Additional file 2: Table S2.** Coding sequences and protein sequences of the identified *GmPME* gene members.

**Additional file 3: Table S3.** The 66 *AtPME* genes in Arabidopsis.

**Additional file 4: Table S4.** Analyses the motifs in soybean PME proteins from the MEME website.

**Additional file 5: Table S5.** Gene density of each chromosome of the soybean genome.

**Additional file 6: Table S6.** The synonymous substitution calculations of soybean homologous genes.

**Additional file 7: Table S7.** The segmentally duplicated gene pairs throughout the soybean genome.

**Additional file 8: Table S8.** The tandemly duplicated gene pairs throughout the soybean genome.

**Additional file 9: Table S9.** Tandemly and segmentally duplicated *GmPME* gene pairs.

**Additional file 10: Table S10.** One-to-one orthologous relationships between the *PME* gene members in *Glycine max* and the other six species.

**Additional file 11: Table S11.** Non-redundant *GmPME* gene IDs associated with the syntenic relationships among soybean and the other six species.

**Additional file 12: Table S12.***Cis*-element analysis of *GmPME* gene promoters.

**Additional file 13: Table S13.** Expression profiles of *GmPME* genes in multiple tissues on Phytozome.

**Additional file 14: Table S14.** Pairwise correlation coefficients between different expressed *GmPME* genes in various tissues.

**Additional file 15: Table S15.** Expression profiles of *GmPME* genes in soybean flower buds from cytoplasmic male sterile line NJCMS1A and its maintainer NJCMS1B.

**Additional file 16: Figure S1.** Phylogenetic expression profiles of *GmPME* genes in soybean flower buds from cytoplasmic male sterile line NJCMS1A and its maintainer NJCMS1B based on the published transcriptome data. The FPKM values were row-scaled with the zero-to-one method.

**Additional file 17: Table S16.** Expression profiles of *GmPME* genes in soybean embryonic axes during seed germination.

**Additional file 18: Figure S2.** Phylogenetic expression profiles of the extracted *GmPME* genes in soybean embryonic axes during seed germination based on the reported transcriptome data. The FPKM values were row-scaled with the zero-to-one method. *GmPME* genes that lacked expression information in the transcriptome data were depicted with the gray color in the heatmap.

**Additional file 19: Table S17.** Sequences of the primers used in this study.

## Data Availability

The reference genome of *Glycine max* used to identify the *PME* genes was released in JGI Phytozome 13 with the accession number of ACUP02000000 (https://phytozome-next.jgi.doe.gov/info/Gmax_Wm82_a2_v1). The genome and its annotation file of *Arabidopsis thaliana* were downloaded from JGI Phytozome 13 (https://phytozome-next.jgi.doe.gov/info/Athaliana_TAIR10). The genome and its annotation file of *Glycine soja* were downloaded from JGI Phytozome 13 (https://phytozome-next.jgi.doe.gov/info/Gsoja_v1_1). The genome and its annotation file of *Vigna unguiculata* were downloaded from JGI Phytozome 13 (https://phytozome-next.jgi.doe.gov/info/Vunguiculata_v1_1). The genome and its annotation file of *Solanum lycopersicum* were downloaded from JGI Phytozome 13 (https://phytozome-next.jgi.doe.gov/info/Slycopersicum_ITAG3_2). The genome and its annotation file of *Oryza sativa* were downloaded from JGI Phytozome 13 (https://phytozome-next.jgi.doe.gov/info/Osativa_v7_0). The genome and its annotation file of *Sorghum bicolor* were downloaded from JGI Phytozome 13 (https://phytozome-next.jgi.doe.gov/info/Sbicolor_v3_1_1). The gene expression data in soybean various tissues were downloaded from Phytozome (https://phytozome.jgi.doe.gov/pz/portal.html). The RNA-Seq reads in flower buds of soybean cytoplasmic male sterile line NJCMS1A and its maintainer NJCMS1B were deposited in Sequence Read Archive database (http://www.ncbi.nlm.nih.gov/Traces/sra/) under the accession number SRP052011. The transcriptome datasets generated in soybean embryonic axis in five time points during germination were downloaded from GEO (https://www.ncbi.nlm.nih.gov/geo/query/acc.cgi?acc=GSE83481) under the accession number GSE83481. Soybean materials used in this study (NJCMS1A, NJCMS1B and Williams 82) were supplied by Soybean Research Institute, National Center for Soybean, Key Improvement Laboratory of Biology and Genetic Improvement of Soybean (General, Ministry of Agriculture), State Key Laboratory of Crop Genetics and Germplasm Enhancement, Jiangsu Collaborative Innovation Center for Modern Crop Production, College of Agriculture, Nanjing Agricultural University, Nanjing 210095, China. All data generated or analyzed during this study are included in this published article and its Additional files.

## Abbreviations

aa: Amino acid
*At*: *Arabidopsis thaliana*
bp: Base pair
CDS: Coding sequence
Chr: Chromosome
CMS: Cytoplasmic male sterility
FPKM: Fragments per kilobase million
*Gm*: *Glycine max*
*GmPME*: *Glycine max PME* gene
*Gs*: *Glycine soja*
HAI: Hours after imbibition
Ka: Non-synonymous substitution
kb: Kilobase
Ks: Synonymous substitution
MW: Molecular weight
NJ: Neighbor-joining
ORF: Opening reading frame
*Os*: *Oryza sativa*
pI: Isoelectric point
PME: Pectin methylesterase
PMEI: PME inhibitor
SAM: Shoot apical meristem
*Sb*: *Sorghum bicolor*
SD: Standard deviation
*Sl*: *Solanum lycopersicum*
UTRs: Untranslated regions
*Vu*: *Vigna unguiculata*
WGD: Whole genome duplication
