# Supplementary figures and images for: Genome-wide identification of PME genes, evolution and expression analyses in soybean (Glycine max L.)
Source: BMC Plant Biol. 2021 Dec 6;21:578. doi: 10.1186/s12870-021-03355-1 (PMC8647493; doi:10.1186/s12870-021-03355-1)

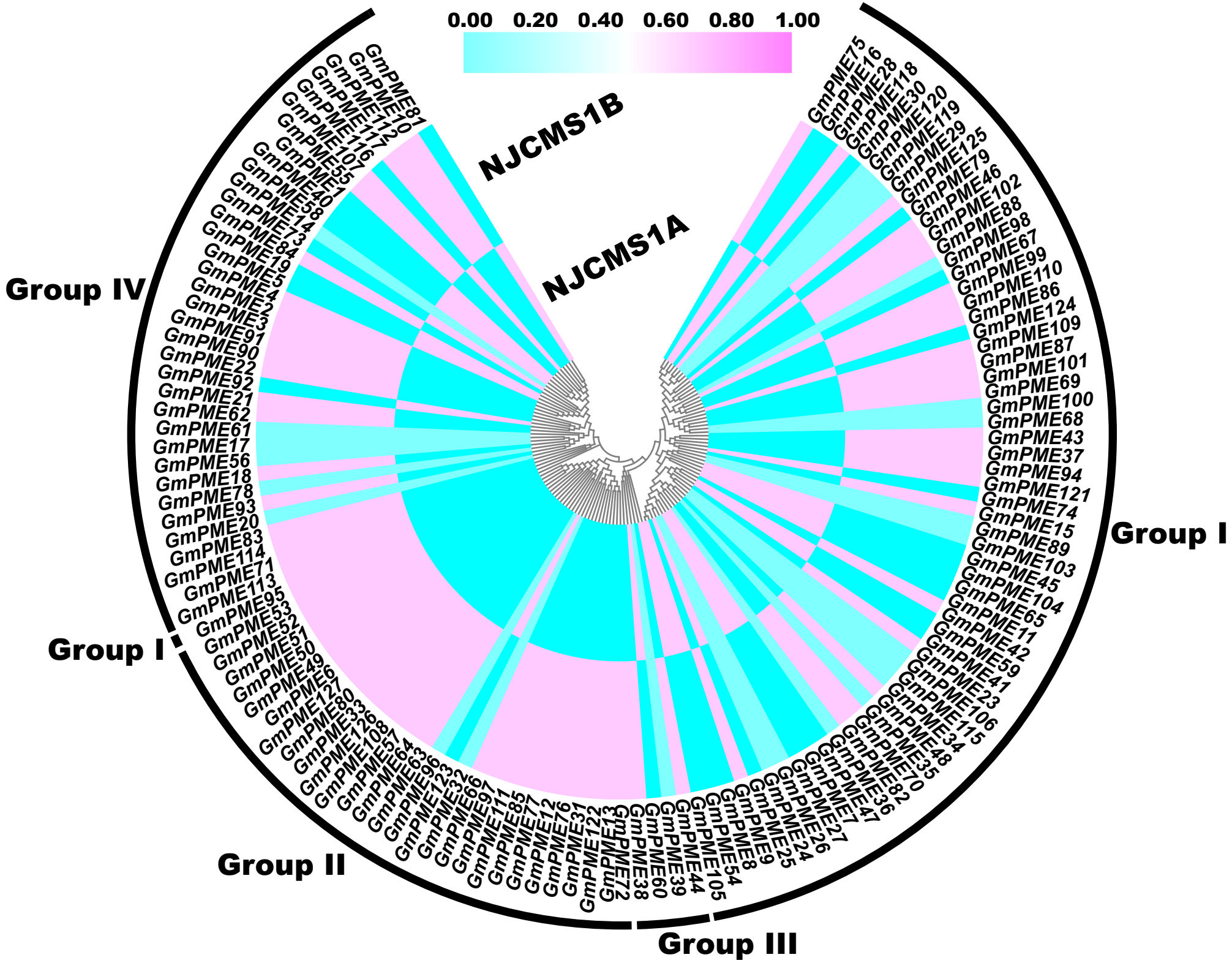

Supplement: Supplementary file 16 — Additional file 16: Figure S1. Phylogenetic expression profiles of GmPME genes in soybean flower buds from cytoplasmic male sterile line NJCMS1A and its maintainer NJCMS1B based on the published transcriptome data. The FPKM values were row-scaled with the zero-to-one method. [file 12870_2021_3355_MOESM16_ESM.pdf]

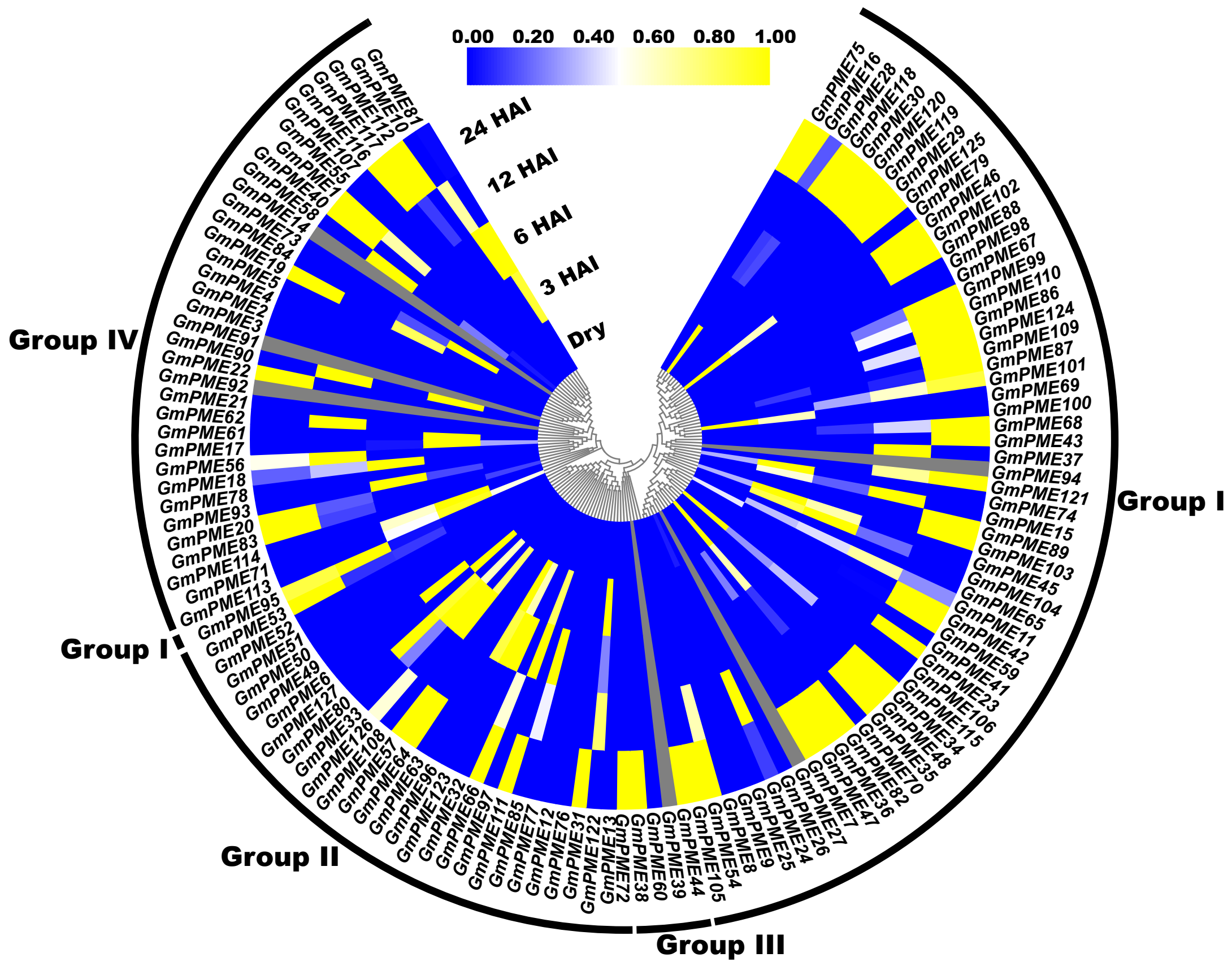

Supplement: Supplementary file 18 — Additional file 18: Figure S2. Phylogenetic expression profiles of the extracted GmPME genes in soybean embryonic axes during seed germination based on the reported transcriptome data. The FPKM values were row-scaled with the zero-to-one method. GmPME genes that lacked expression information in the transcriptome data were depicted with the gray color in the heatmap. [file 12870_2021_3355_MOESM18_ESM.pdf]
